# Systematic review of pharmacotherapy for atypical facial pain: evaluation of pain reduction, depression, anxiety and quality of life

**DOI:** 10.1080/07853890.2025.2476050

**Published:** 2025-03-14

**Authors:** Abdullah F. Alshammari, Hind M. Alassaf, Ahmed A. Madfa, Sattam S. Alshammari, Sameer Shaikh, Hassan H. Abed, Ali A. Alqarni, Freah L. Alshammary, Khlood A. Alkurdi

**Affiliations:** a Department of Basic Dental and Medical Science, College of Dentistry, University of Ha’il, Ha’il, Kingdom of Saudi Arabia; b School of Nursing and Midwifery, Queen’s University Belfast, Belfast, United Kingdom; c Department of Restorative Dental Science, College of Dentistry, University of Ha’il, Ha’il, Kingdom of Saudi Arabia; d Department of Preventive Dental Science, College of Dentistry, University of Ha’il, Ha’il, Kingdom of Saudi Arabia; e Department of Maxillofacial Surgery and Diagnostic Science, College of Dentistry, University of Ha’il, Ha’il, Kingdom of Saudi Arabia; f Department of Basic and Clinical Oral Sciences, Faculty of Dentistry, Umm Al-Qura University, Makkah, Kingdom of Saudi Arabia; g Department of Oral and Maxillofacial Surgery and Diagnostic Sciences, Faculty of Dentistry, Taif University, Taif, Kingdom of Saudi Arabia; h Department of Preventive Dentistry, College of Dentistry, University of Ha’il, Ha’il, Kingdom of Saudi Arabia; i Ministry of Health, Qassim Health Cluster, King Saud Hospital, Unayzah, Kingdom of Saudi Arabia; j Institute of Dentistry, Queen Mary University of London, London, United Kingdom

**Keywords:** Atypical facial pain, pharmacotherapy, pain management, quality of life, systematic review, antidepressants, anticonvulsants, neuromodulators

## Abstract

**Background/purpose:**

Atypical facial pain (AFP) is a chronic condition characterized by persistent facial pain without clear clinical signs, making diagnosis and treatment difficult. Common pharmacological treatments include antidepressants, anticonvulsants and neuromodulators, but their effectiveness remains uncertain, necessitating a systematic review to guide clinical practice.

**Materials and methods:**

Following PRISMA 2020 guidelines, randomized controlled trials (RCTs) evaluating pharmacological treatments for AFP in adults were included. A comprehensive search of five databases without date restrictions was performed. Data on pain reduction, quality of life, and adverse events were extracted. The Cochrane Risk of Bias (RoB 2.0) tool assessed bias, and evidence quality was evaluated using the Critical Appraisal Skills Programme tool.

**Results:**

Out of 196 studies identified, 10 RCTs met the inclusion criteria. Pharmacological responses varied significantly across studies. Antidepressants such as dothiepin and clomipramine demonstrated significant pain reduction, whereas venlafaxine showed limited efficacy. However, onabotulinum toxin A and sumatriptan exhibited inconsistent or minimal effects on AFP pain intensity. Adverse events were reported across multiple treatments, ranging from mild side effects such as dry mouth and nausea to severe reactions like diplopia and facial asymmetry, which impacted adherence. Despite some positive outcomes, the heterogeneity in study methodologies, outcome measures, and follow-up durations limited direct comparisons between interventions.

**Conclusion:**

This systematic review highlights the mixed efficacy of pharmacological treatments for AFP, with certain medications demonstrating superior pain relief in specific patient subgroups. Given the variability in response and adverse events, a multimodal approach combining pharmacological and non-pharmacological therapies may offer the most effective management strategy. Future research should focus on standardized treatment protocols, long-term efficacy, and personalized treatment plans to optimize patient care.

## Introduction

Atypical facial pain (AFP) is a chronic and often debilitating condition characterized by persistent, unexplained facial pain. Unlike other facial pain syndromes such as trigeminal neuralgia, AFP lacks distinct clinical signs, making its diagnosis and management challenging. The pain associated with AFP is typically constant, dull, or burning and can significantly impair the quality of life of those affected [1].

Furthermore, there is growing recognition of the strong association between AFP/PIFP and psychiatric comorbidities. Studies suggest that up to 50% of patients with chronic orofacial pain, including AFP, experience significant depressive or anxiety symptoms [2]. Contributing to emotional distress, social withdrawal, and decreased overall well-being, reinforcing a cycle of pain and psychological burden [3]. Despite its prevalence, the aetiology of AFP remains poorly understood, and its idiopathic nature further complicates its treatment. Patients with AFP often experience significant distress and disability, underscoring the need for effective therapeutic strategies [1,4].

The complex and multifactorial nature of AFP has led to the development of a wide range of pharmacological and nonpharmacological treatment approaches [5]. Pharmacological treatments are often the first line of defense and include various classes of medications such as antidepressants, anticonvulsants, and neuromodulators. These medications are prescribed based on their proposed mechanisms of action in the pain pathways involved in AFP. However, the heterogeneity of the patient population and the subjective nature of pain present challenges in evaluating the efficacy of these treatments [6]. The variability in the response to pharmacological interventions further emphasizes the need for personalized treatment plans.

Antidepressants, particularly tricyclic antidepressants (TCAs) such as amitriptyline, have been widely used to manage AFP because of their analgesic properties. These medications are believed to modulate pain through their effects on neurotransmitters such as serotonin and norepinephrine [5]. Similarly, anticonvulsants such as carbamazepine and gabapentin are employed because of their ability to stabilize nerve cell membranes and reduce abnormal electrical activity in the brain, which may contribute to pain. Neuromodulators, including onabotulinum toxin A (Botox), have also been investigated for their potential to alleviate pain by inhibiting neurotransmitter release and reducing muscle contraction. Despite their theoretical benefits, the clinical efficacy of these treatments varies, and their adverse effects often limit their use [3].

Anticonvulsants, another key class of drugs used in AFP management, stabilize neuronal membranes and prevent the abnormal firing of nerve signals that may contribute to pain. Medications such as carbamazepine and gabapentin have shown varying degrees of efficacy in clinical studies. For instance, carbamazepine is known for its effectiveness in treating trigeminal neuralgia, a related condition; however, its application in AFP is unclear. In contrast, gabapentin is favored for its relatively mild side-effect profile, making it a viable option for long-term management [3].

Neuromodulators, such as onabotulinum toxin A (Botox), represent a more recent addition to the pharmacological arsenal against AFP. Botox, primarily used in cosmetic applications, has been studied for its pain-relieving properties. It inhibits the release of the neurotransmitters involved in pain transmission, thereby reducing muscle contraction and potentially alleviating pain [7]. Clinical trials investigating Botox have shown promise, although the results are not universally consistent. Further research is needed to establish standardized treatment protocols.

In addition to pharmacological treatment, the role of non-pharmacological interventions cannot be overlooked. Nonpharmacological interventions such as cognitive behavioral therapy (CBT), physical therapy, and acupuncture are also used in the management of AFP. These approaches address the psychological and physical aspects of pain, promote coping strategies, and improve functional outcomes [2,8]. Physical therapy and acupuncture are other nonpharmacological modalities that can complement pharmacological treatments and offer a holistic approach to manage AFP. However, the integration of nonpharmacological therapies into standard care remains inconsistent, and their effectiveness is often evaluated in conjunction with pharmacological treatments. The combination of these approaches reflects the need for a comprehensive and multidisciplinary strategy to manage AFP.

Despite various treatment options, the lack of high-quality evidence complicates clinical decision-making for AFP. Variability in treatment responses highlights the need for a personalized approach. This systematic review evaluates the efficacy of pharmacological treatments through data synthesis from randomized controlled trials (RCTs), aiming to identify the most effective interventions for pain reduction and quality of life improvement. By critically assessing existing literature, this review provides evidence-based recommendations to guide clinical practice, addressing the key PICO question (patient, intervention, comparison, outcome): ‘In adults with atypical facial pain, how effective are pharmacological treatments compared to placebo or other pharmaceutical interventions in reducing pain intensity and improving quality of life?’

## Materials and methods

### Eligibility criteria

This systematic review was performed according to the Preferred Reporting Items for Systematic Reviews and Meta-Analyses (PRISMA) 2020 guidelines. The review was conducted in accordance with a pre-specified protocol, which was registered with PROSPERO (registration number: CRD42024573872).

Only randomized controlled trials (RCTs) were included. The study population consisted of adult patients (aged ≥18 years) diagnosed with AFP were also included. No restrictions were imposed on the type, dosage, or route of administration of the interventions, which were compared with placebo or alternative interventions. All included studies were published in English.

### Information sources and search strategy

A thorough search was performed across five electronic databases: PubMed, Embase (Excerpta Medica database), CENTRAL (Cochrane Central Register of Controlled Trials), Scopus, and Google Scholar. The search was performed without date restrictions to retrieve all available evidence and reach comprehensive conclusions. The search terms and strategies used included combinations of the following MeSH terms and keywords: ‘Randomized Controlled Trial’ OR ‘RCT’, ‘Atypical Facial Pain’ OR ‘Persistent Idiopathic Facial Pain’, ‘Facial Pain’ OR ‘Chronic Facial Pain’, ‘Pain Management’ OR ‘Pain Treatment’ OR ‘Therapeutics’, ‘Pharmacological Interventions’ OR ‘Drug Therapy’ OR ‘Pharmaceutical Preparations’, and ‘Analgesics’ OR ‘Antidepressants’ OR ‘Anticonvulsants’ OR ‘Neuromodulators’.

Two authors, AA and KA independently conducted each step of the search process to ensure that all necessary studies were retrieved. Both authors conducted a ‘snowball’ search to identify additional studies by reviewing the reference lists of publications deemed eligible for full-text review and utilizing Google Scholar to identify and screen studies that cited these publications.

### Selection process and data collection process

The titles and abstracts of all the retrieved studies were independently reviewed by three researchers (AA, KA, and AM), and discrepancies were resolved by discussion until a consensus was reached. Subsequently, researchers working in pairs independently screened the titles and abstracts of the studies. Two researchers (AA and KA) then independently conducted full-text screening for study inclusion. In cases of disagreement during full-text screening, consensus on inclusion or exclusion was reached through discussion, with a third researcher (AM) consulted, if necessary, to make the final determination.

### Data items

The primary outcome measures were reduction in pain intensity and improvement in quality of life among patients with AFP. In instances for which composite outcomes were not provided, the corresponding authors were contacted to request supplementary information, including primary data. The extracted data encompassed various categories, including study characteristics. Details of the interventions, including dose, route, and timing of administration, were meticulously recorded.

Primary outcome measures were categorized into three main areas: pain reduction, quality of life improvement, and adverse events. Secondary outcome includes acute pain management following injection.

The two reviewers compared the extracted data, and any discrepancies were resolved through discussion. The data were then entered into Review Manager 5 software (Review Manager 2014) by AA and double-checked to ensure accuracy. In cases with unclear information, the authors of the original reports were contacted for further clarification.

### Study risk of bias assessment

The risk of bias in the included studies was assessed using the revised Cochrane risk-of-bias tool for randomized trials (RoB 2.0), developed by the University of Bristol, with supplementary guidance for cluster-randomized and crossover trials. Two independent reviewers (AA and KA) applied the tool to each included study, systematically documenting the supporting information and rationales for the risk of bias judgments across all domains (low, high, and some concerns). Any discrepancies in these judgments or their justifications were resolved through discussion to achieve a consensus, with a third reviewer (AM) serving as an arbitrator, if necessary.

### Quality assessment

Two reviewers (AA and KA) independently assessed the certainty of the evidence. The Critical Appraisal Skills Programme (CASP) tool was used to evaluate the quality of the included studies. Decisions were justified to categorize the certainty of the evidence using detailed footnotes, and comments were provided to help readers understand the results and their implications. The assessments were recorded systematically to ensure transparency and reproducibility.

### Synthesis methods

If the data exhibited heterogeneity, a descriptive analysis was employed to comprehensively summarize and characterize the dataset. Conversely, if the data were deemed comparable, appropriate statistical methods were applied to facilitate robust analysis and inferential conclusions. The statistical techniques were selected based on the specific characteristics of the data and research objectives, ensuring a rigorous evaluation and valid interpretation of the findings.

## Results

### Study selection

**Figure 1.**
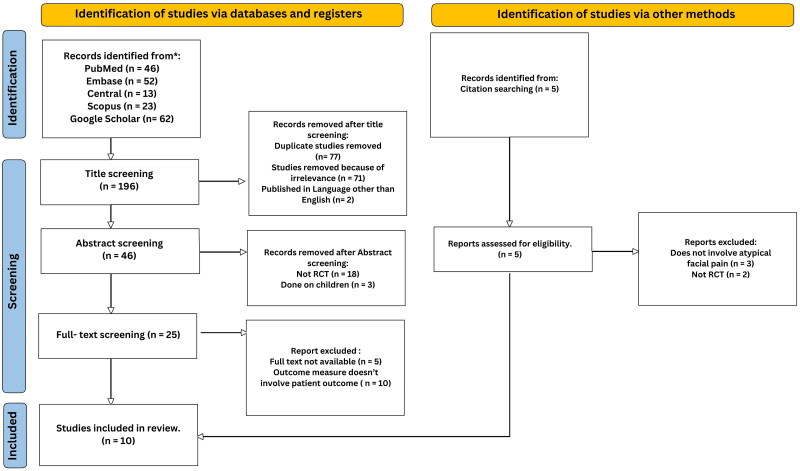
PRISMA flowchart illustrating the systematic search and selection process.

After searching the five databases, 196 studies were identified. After title screening, 150 studies were excluded, leaving 46 for abstract screening. After abstract screening, 21 studies were excluded, leaving 25 for full-text screening. After full-text screening, ten studies met the primary inclusion criteria and were eligible for inclusion in this review (Figure 1).

### Quality and risk of bias assessment

The quality assessment of the included studies, as summarized in Table 1, demonstrated variability in methodological rigor across different domains. Most studies, including those by Jamtoy et al. (2023), List et al. (2006), and Forssell et al. (2004), exhibited strong adherence to study design validity, with clearly defined research questions and the implementation of randomization techniques. Additionally, blinding and baseline comparability were well-addressed in several studies, such as List et al. (2006) and Sharav et al. (1987), enhancing the reliability of the reported treatment effects. However, notable limitations were identified in the reporting of outcomes and study applicability. Missing outcome data was a common concern, particularly in studies like Jamtoy et al. (2023) and Forssell et al. (2004), often due to attrition at follow-ups and the absence of advanced statistical techniques such as multiple imputation or mixed-level modelling to account for incomplete data. Furthermore, the external validity of several studies, including Harrison et al. (1997) and Al Balawi et al. (1996), was questioned, as generalizability and the practical relevance of interventions were frequently categorized as ‘Cannot tell’ in the appraisal. Older studies, such as Feinmann et al. (1983) and Nicol et al. (1969), demonstrated weaker reporting practices, whereas more recent studies adhered more closely to methodological rigor, particularly in the precision of confidence intervals and risk-benefit evaluations. To enhance transparency, Table 1 has been supplemented with a footnote detailing the specific questions within each domain, allowing for a clearer interpretation of the quality assessment criteria.

**Table 1.** Quality assessment results.

Author											
	Section A*			Section B*			Section C*			Section D*	
	Q1	Q2	Q3	Q4	Q5	Q6	Q7	Q8	Q9	Q10	Q11
Jamtoy et al. (2023) [9]	Yes	Yes	Yes	Yes	Yes	Yes	Yes	Yes	No	Cannot tell	Cannot tell
List et al. (2006) [10]	Yes	Yes	Yes	Yes	Yes	Yes	Yes	Yes	Yes	Yes	Yes
Forssell et al. (2004) [11]	Yes	Yes	No	Yes	Cannot tell	Yes	Yes	Yes	Cannot tell	Yes	Cannot tell
Harrison et al. (1997) [12]	Yes	Yes	Yes	Yes	Cannot tell	Yes	Yes	Yes	No	Yes	Cannot tell
Al Balawi et al. (1996) [13]	Yes	Yes	No	Yes	Yes	Yes	Yes	Yes	Cannot tell	Yes	Cannot tell
Loldrup et al. (1989) [14]	Yes	Yes	No	Yes	Yes	Yes	Yes	Yes	Yes	Yes	Yes
Marbach et al. (1988) [15]	Yes	Yes	No	Yes	Yes	Yes	Yes	Yes	Cannot tell	Yes	Yes
Sharav et al. (1987) [16]	Yes	Yes	No	Yes	Yes	Yes	Yes	Yes	Yes	Yes	Yes
Feinmann et al. (1983) [17]	Yes	Yes	Yes	Yes	Cannot tell	Yes	Yes	Yes	Yes	Yes	Yes
Nicol et al. (1969) [18]	Yes	Yes	No	Yes	Yes	Yes	Yes	Yes	Yes	Yes	Yes

**Domain:**.

**Section A** (Study Design Validity): **Q1** – Clear research question, **Q2** – Randomization, **Q3** – Follow-up.

**Section B** (Methodology & Treatment): **Q4** – Blinding, **Q5** – Baseline comparability, **Q6** – Equal treatment.

**Section C** (Results & Interpretation): **Q7** – Reporting of intervention effects, **Q8** – Precision & confidence intervals, **Q9** – Risk-benefit evaluation.

**Section D** (Applicability & Relevance): **Q10** – Generalizability, **Q11** – Practical value of the intervention.

**Figure 2.**
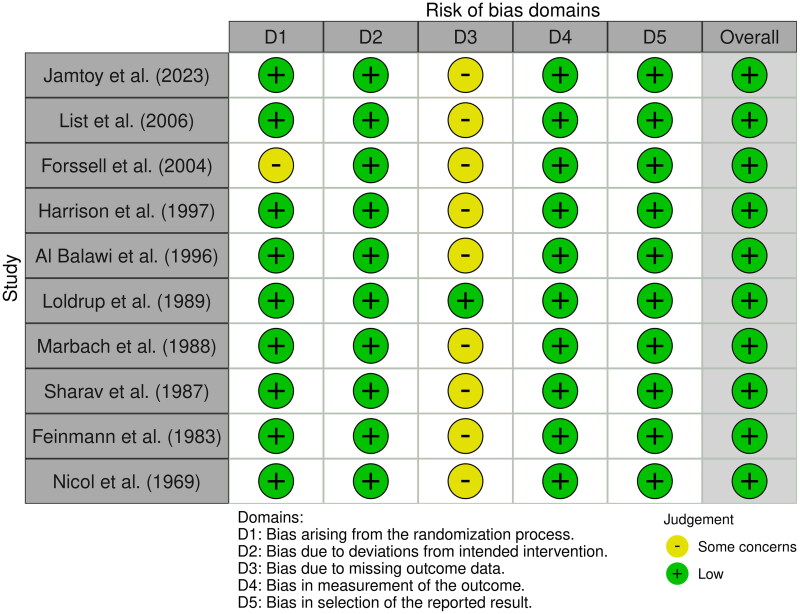
Risk of bias assessment.

The risk of bias assessment of the included studies, presented in Figure 2, illustrates the variation in methodological rigor across different domains of the RoB-2 tool. While studies such as List et al. (2006), Sharav et al. (1987), and Feinmann et al. (1983) demonstrated low risk of bias across multiple domains, several studies exhibited concerns in specific areas. Notably, Forssell et al. (2004), Harrison et al. (1997), and Jamtoy et al. (2023) showed concerns in the randomization process (D1) due to insufficient details on allocation procedures. Additionally, missing outcome data (D3) was a recurrent issue, particularly in Forssell et al. (2004), Harrison et al. (1997), and Marbach et al. (1988), where follow-up attrition and the absence of modern statistical techniques, such as multiple imputation, contributed to a higher risk classification.

**Figure 3.**
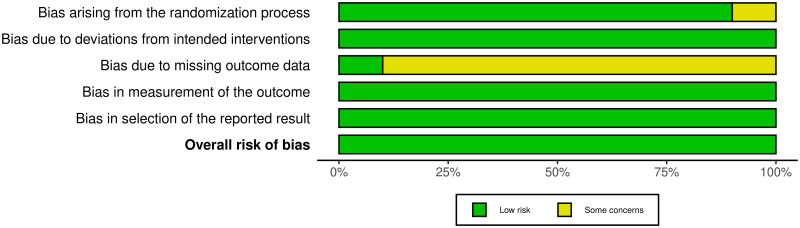
Overall risk of bias.

The overall risk of bias is summarized in Figure 3, demonstrating that while a proportion of studies, including List et al. (2006) and Sharav et al. (1987), maintained a low risk of bias, many exhibited some concerns or high risk, particularly in deviations from intended interventions (D2) and bias in the measurement of outcomes (D4). Studies such as Al Balawi et al. (1996) and Jamtoy et al. (2023) showed inconsistencies in adherence to intended interventions, while Harrison et al. (1997) and Forssell et al. (2004) had unclear blinding procedures, potentially influencing outcome assessments. In contrast, bias in the selection of reported results (D5) was less frequently observed, with studies such as Nicol et al. (1969) and Feinmann et al. (1983) demonstrating adherence to appropriate reporting standards.

### Results of individual studies

Patient characteristics were thoroughly examined for all ten included studies, which were all double-blinded RCTs, except one study, which was a triple-blind RCT (where condition assignment is hidden from participants, experimenters and researcher who analysing the data) conducted by Jamtoy et al. (2023) [9]. All but one were national-based studies, conducted in Norway (*n* = 1), Sweden (*n* = 1), Finland (*n* = 1), the United Kingdom (UK) (*n* = 3), Denmark (*n* = 1), and the United States of America (USA) (*n* = 2). Only one study involved a collaboration between two countries, USA and Israel (*n* = 1).

For the present study, narrative data synthesis was considered the best approach to synthesize results, as a meta-analysis could not be performed owing to heterogeneity. This is because the included studies used different intervention types, doses, routes of administration (injections and tablets), and pain assessment tools (such as the McGill pain questionnaire [MPQ] and visual analogue scale [VAS]).

The included studies used a variety of interventions for treating AFP: in four of the studies, the intervention group received antidepressants including clomipramine, amitriptyline, dothiepin, and venlafaxine [11,14,16,17]. In two studies, the intervention group received sumatriptan [12,13]. In the study by Jamtoy et al. (2023), the intervention group received botulinum toxin A [9]. In the study by List et al. (2006), the intervention group received lidocaine [10], while in the study by Nicol et al. (1969) study, the intervention group received an anticonvulsant (carbamazepine) [18]. The route of administration varied. In four of the included studies, the participants received the intervention as a tablet [11,14,16,19] whereas in six of the included studies, the participants received the intervention as a subcutaneous injection [9,10,12,13,15,17]. The included studies used a various placebo treatments, in the studies by by Sharav (1987) [16], Nicol (1969) [18], Forssell (2004) [11], and Feinmann (1983) [17] placebo treatment consisted of identically appearing tablets that was administered in the same manner as the treatment in the intervention group. Where other studies used placebo injections, such as normal saline solution or 0.47% lidocaine as in Marbach (1988) [15] and List (2006) [10] studies, while Balawi (1996) [13] used saline as a placebo. In the study by Jamtøy (2023) [9], the placebo consisted of 25 Allergan-units of Botox suspended in 0.5 ml of isotonic saline or 0.5 ml of saline alone. Some studies did not specify the exact nature of their placebo treatments, including those by Loldrup (1989) [14] and Harrison (1997) [12].

To assess pain intensity, five studies used the VAS [10,11,14–16]. The VAS is a widely used tool in clinical settings for pain assessment, where it is a subjective measure to quantify the intensity of pain experienced by an individual. A handwritten mark on a 10-cm line representing a continuum between ‘no pain’ and ‘worst pain’ is used to record scores. Three studies used the MPQ [9,12,13], which is an tool used to assess and quantify pain. The MPQ includes four main measures, namely sensory, affective, evaluative, and miscellaneous. Feinmann et al. (1983) recorded the intensity and frequency of pain on a 0 to 4 scale, where 1 = mild, occasional; 2 = moderate, frequent; 3 = appreciable, frequent; and 4 = severe, constant [17]. Nicol et al. (1969) assessed pain intensity as follows: excellent (absence or only slight hints of pain), good (pain still present but considerably decreased), poor (slight improvement in pain frequency or intensity), and unchanged (no pain noted) [18]. Table 2 summarizes the main characteristics and the diagnostic criteria of the included studies.

**Table 2.** Characteristics of the included studies.

Author/ Country	Study design	Participant		Intervention		Diagnostic criteria	Outcome	Main findings
		I	C	I	C			
Jamtoy et al. (2023)^9^ Norway	Triple-blind, cross-over RCT	**13**	**14**	25 units of botulinum toxin A sphenopalatine ganglion (SPG).	Placebo	ICHD-3 criteria for PIFP	Pain severity, anxiety, depression, and adverse effects	There was no statistically significant difference in pain reduction between BTA and placebo in weeks 5-8.
List et al. (2006)^10^ Sweden	Double-blind, cross-over RCT	**35**	**35**	1.5 ml of either 20 mg/ml lidocaine with 12.5 µg/ml adrenaline local injections	Placebo	Exclusion-based diagnosis of AFP	Pain severity Adverse effects	Lidocaine provided significant but incomplete pain relief compared to placebo.
Forssell et al. (2004)^11^ Finland	Double-blind, cross-over RCT	**11**	**9**	75 mg of venlafaxine tablets	Placebo	Exclusion-based diagnosis	Pain severity, anxiety, depression, and adverse effects	Venlafaxine was modestly effective in treating AFP, showing no significant difference in pain intensity reduction compared to placebo.
Harrison et al. (1997)^12^ UK	Double-blind, cross-over RCT	**17**	**17**	6 mg subcutaneous sumatriptan	Placebo	No standardized criteria reported	Pain severity	Sumatriptan significantly reduced pain scores at 120 minutes post-injection compared to placebo but was not considered clinically effective for AFP due to the temporary nature of pain relief.
Al Balawi et al. (1996)^13^ UK	Double-blind, cross-over RCT	**18**	**19**	6 mg sumatriptan subcutaneous Injection	Placebo	McGill Pain Questionnaire classification	Pain severity Adverse effects	Sumatriptan significantly reduced sensory, affective, and total pain scores at 120 minutes post-injection compared to placebo.
Loldrup et al. (1989)^14^ Denmark	Double-blind RCT	**84**	87	Up to 150 mg/day clomipramine tablets	Placebo	Chronic idiopathic pain syndrome classification	Pain severity, anxiety and depression	Clomipramine was more effective in patients with depression, both clomipramine and mianserin were effective for tension headache, and placebo was more effective for low back pain.
Marbach et al. (1988)^15^ USA	Double-blind, cross-over RCT	**24**	**21**	4% lidocaine hydrochloride	Placebo	Deafferentation neuralgia and myofascial pain classification	Pain severity Adverse effects	Intranasal cocaine provided significant pain relief at higher concentrations, with the 40% solution showing the greatest analgesic effect, while lidocaine had minimal impact on pain relief.
Sharav et al. (1987)^16^ USA/Israel	Double-blind, cross-over RCT	**11**	**9**	50 to150 mg amitriptyline tablets	Placebo	Minimum 6-month duration, exclusion-based diagnosis	Pain severity and depression	Amitriptyline significantly reduced chronic oral-facial pain compared to placebo, with the analgesic effect being independent of its antidepressant properties.
Feinmann et al. (1983)^17^ UK	Double-blind RCT	**25**	**20**	25 up to 150 mg dothiepin plus use of nocturnal biteguard	Placebo	Psychogenic facial pain classification	Pain severity Adverse effects	Dothiepin was significantly more effective than placebo in achieving pain relief, with 71% of patients in the dothiepin group pain-free at nine weeks compared to 47% in the placebo group.
Nicol et al. (1969)^18^ USA	Double-blind RCT	**20**	**7**	200 mg carbamazepine tablet	Placebo	Atypical facial neuralgia classification	Pain severity Adverse effects	Carbamazepine provided good to excellent pain relief in 73% of patients, while 27% had poor results or no change.

I = Intervention group, C = Control group, BTA = onabotulinum toxin A, RCT = Randomized controlled trial, USA = United States of America, UK = United Kingdom, visual analog scale = VAS.

### Pain intensity

The evaluation of pain intensity reduction across multiple studies revealed the varying effectiveness of different pharmacological treatments in managing AFP. In the study by Jamtoy et al. (2023), treatment with onabotulinum toxin A (BTA) produced no significant reduction in average pain intensity compared to placebo in weeks 5–8 (mean difference = 0.00; 95% CI = −0.57 to 0.57) [9]. However, post hoc analyses suggested potential early pain reduction within the first four weeks post-injection. Similarly, Forssell et al. (2004) reported no significant difference in pain intensity reduction with venlafaxine compared to placebo over a 10-week period (*p* = 0.64) [11]. Conversely, Harrison (1997) found that subcutaneous administration of sumatriptan significantly reduced pain scores at 120 min post-injection, although the reduction was considered too small for clinical benefit (*p* = 0.153) [12]. Moreover, Loldrup et al. (1989) observed that clomipramine and mianserin did not significantly reduce pain intensity compared to a placebo in the overall sample. However, clomipramine was effective in patients with depression (*p* < 0.05) [14]. Furthermore, Marbach et al. (1988) found significant pain reduction with intranasal cocaine, particularly at higher concentrations (*p* < 0.05), whereas lidocaine did not show significant changes compared to placebo [15]. In addition, Feinmann et al. (1983) demonstrated that dothiepin significantly reduced pain intensity in patients with psychosomatic facial pain, with 71% of patients being pain-free at nine weeks compared to 47% with placebo (*p* < 0.05) [17]. Finally, Nicol (1969) study, six patients were diagnosed with atypical facial neuralgia (now classified under AFP/PIFP). Among these, only two were included in the final analysis, with one patient reporting partial pain relief and the other showing no significant improvement with carbamazepine [18].

### Depression

The impact of pharmacological treatments on depression varied across studies, with some treatments showing significant benefits while others did not. In the study by Forssell et al. (2004), venlafaxine did not significantly reduce depression scores compared to placebo (*p* = 0.16) [11]. Similarly, Harrison (1997) did not specifically address depression as an outcome measure in a study of sumatriptan [12]. In contrast, Loldrup et al. (1989) found that clomipramine significantly reduced depressive symptoms in patients with major depression (*p* < 0.05), indicating its dual efficacy in managing pain and depression [14]. Furthermore, Sharav et al. (1987) reported that amitriptyline reduced depression scores in the depressed group (*p* < 0.01) but had no effect on nondepressed patients, highlighting its specific impact on depressive symptoms [16]. Moreover, Feinmann et al. (1983) showed that dothiepin was not superior to placebo in relieving depression, although both treatments led to significant improvements in depression scores (*p* < 0.01) [17].

### Anxiety

Anxiety levels were not a primary focus of most studies; therefore, data on this domain are limited. However, Loldrup et al. (1989) noted that mianserin produced the best outcomes in patients with major anxiety syndrome, although the difference was not statistically significant when compared to clomipramine and placebo [14]. In contrast, Harrison (1997) and Marbach et al. (1988) did not explicitly measure anxiety but noted indirect reductions in anxiety based on other observed outcomes [12,14].

### Follow-up

The follow-up periods and their outcomes varied significantly among the studies. For instance, Jamtoy et al. (2023) structured a 12-week post-injection evaluation period, followed by an eight-week washout, indicating a possible longer-lasting effect of BTA beyond the initial 12 weeks [9]. Similarly, Forssell et al. (2004) conducted regular assessments over a 10-week period but did not find significant differences in pain intensity reduction between venlafaxine and placebo (*p* = 0.64) [11]. Moreover, Harrison (1997) included follow-ups at 60- and 120-minutes post-injection, showing temporary pain relief with sumatriptan (*p* < 0.05) [12]. In addition, Loldrup et al. (1989) conducted a six-week follow-up, highlighting the need for extended treatment to observe significant effects (*p* < 0.05) [14].

Sharav et al. (1987) included one-week and four-week assessments with a two-week washout period, and significant pain relief with amitriptyline was observed only after four weeks (*p* < 0.01) [16]. Feinmann et al. (1983) conducted a 12-month follow-up and showed that 81% of patients were pain-free at the end of the study period, although some experienced recurrence after discontinuing medication (*p* < 0.01) [17]. Finally, the study of Nicol (1969), which had a follow-up period of up to 46 months, demonstrated sustained pain relief with carbamazepine in most patients (*p* < 0.05) [18].

### Need for painkillers after injection

The need for additional painkillers varied across studies, reflecting the efficacy of the primary treatments. For example, in the study by Jamtoy et al. (2023), ten participants required additional painkillers on the day of injection, indicating the need for acute pain management (*p* < 0.05) [9]. Forssell et al. (2004) reported minimal analgesic use, suggesting that lidocaine provided sufficient pain relief during the study period (*p* = 0.56) [11]. Harrison (1997) found no significant differences in the need for rescue medication between sumatriptan and placebo groups [12]. Additionally, Sharav et al. (1987) did not highlight specific differences in the need for additional painkillers during treatment with amitriptylin [16]. However, Feinmann et al. (1983) reported an 83% reduction in analgesic use in the dothiepin group compared to 42% in the placebo group at nine weeks (*p* < 0.01), indicating a significant reduction in the need for additional painkillers [17]. In contrast, Nicol (1969) did not specifically address the need for additional painkillers but instead focused on the efficacy of carbamazepine as the primary analgesic [18].

### Adverse events

Only four of the included studies reported the adverse events experienced by the participants. In the study by Jamtoy et al. (2023), six adverse events were reported in the intervention group: diplopia (*n* = 3), facial asymmetry (*n* = 2), retroorbital pain (*n* = 2), nasolabial fold asymmetry (*n* = 1), pain or swelling (*n* = 1), and difficulty holding the head upright (*n* = 1). Jamtoy et al. (2023) observed two adverse events in the control group: retroorbital pain (*n* = 1) and pain or swelling (*n* = 1) [9]. List et al. (2006) also reported three adverse events due to the use of lidocaine, including headaches, increased pain, and heart palpitations; five adverse events were reported in the control group, including headache, increased pain, dizziness, tiredness, and paresthesia. However, the authors did not report the number of participants who experienced these events [10].

Forssell et al. (2004) reported ten adverse events experienced by participants in both groups. The reported adverse events in the intervention group included difficulty urinating (*n* = 9), fatigue (*n* = 18), loss of appetite (*n* = 15), nausea (*n* = 12), dry mouth (*n* = 12), constipation (*n* = 11), sweating (*n* = 18), nightmares (*n* = 11), headache (*n* = 18), and palpitations (*n* = 14). The reported adverse events in the control group included difficulty urinating (*n* = 6), fatigue (*n* = 17), loss of appetite (*n* = 14), nausea (*n* = 11), dry mouth (*n* = 12), constipation (*n* = 10), sweating (*n* = 18), nightmares (*n* = 9), headaches (*n* = 17), and palpitations (*n* = 13) [11].

Adverse events were also observed by Al Balawi et al. (1996) in their study. In the intervention group, these events included neurological disorders: migraine (*n* = 1), drowsiness/sedation (*n* = 1), dizziness/vertigo (*n* = 2), tremors (*n* = 1), tingling (*n* = 1), warm/hot sensation (*n* = 5), headache (*n* = 7), and facial pain (*n* = 1); cardiovascular disorders: palpitations (*n* = 1); ear, nose, and throat disorders: nasal cavity/sinuses (*n* = 1) and throat symptoms (*n* = 1); gastrointestinal disorders: nausea and/or vomiting (*n* = 2), dysphagia (*n* = 1), and mouth disorders (*n* = 5); musculoskeletal disorders: neck pain/stiffness (*n* = 3), backache (*n* = 1), and feeling of tightness (*n* = 1); respiratory disorders: dyspnea (*n* = 1); and miscellaneous disorders: discomfort (*n* = 1), pressure sensation (*n* = 1), feeling of heaviness (*n* = 6), jaw symptoms (*n* = 1), and injection site reaction (*n* = 12). The reported events in the control group included neurological disorders, such as migraine (*n* = 1) and headache (*n* = 1), and miscellaneous disorders, such as discomfort (*n* = 1) [13].

## Discussion

This systematic review offers an extensive assessment of the effectiveness of pharmacotherapy in treating AFP, concentrating on alleviating pain, depression, anxiety, and quality of life. The results indicate that pharmacological treatment, especially antidepressants and anticonvulsants, can greatly lower pain levels and enhance quality of life in AFP patients. Nonetheless, the findings about the effects on depression and anxiety are not as definitive, emphasizing the necessity for additional research in this field.

The assessment of decreases in pain intensity in various studies has shown diverse outcomes regarding the efficacy of various drugs for treating AFP. The credibility and accuracy of pain evaluation instruments validate their effectiveness in gauging pain levels and reactions to therapy. Five studies used the VAS [10,11,14–16] to evaluate pain severity. The VAS is commonly used for assessing the severity of pain and is typically shown as a 100 mm line with ends indicating the highest and lowest levels of pain [11,20]. This technique is preferred owing to its simplicity and ability to identify subtle differences in pain perception [11]. Three studies highlighted the effectiveness of the MPQ as a comprehensive tool for assessing pain. Taking this viewpoint into account is crucial for assessing the effectiveness of pharmacological treatments, as it offers a more comprehensive understanding of patient experiences and results.

The variety of therapies employed in these investigations emphasize the need for a multimodal strategy to effectively manage AFP [11,14,16,17]. The distinct modes of action of venlafaxine, sumatriptan, BTA, clomipramine, maniaserin, dothiepine, carbamazepine, and lidocaine can be advantageous in various aspects of pain modulation. The particular clinical presentation of AFP, patient’s reaction, and comorbid disorders may influence intervention decisions. Antidepressants and sumatriptan mainly alter neurotransmitter levels and vascular responses, whereas lidocaine relieves localized pain and BTA targets muscle components. Further investigations and comparison studies are required to determine the best treatment plan for AFP [11,14,16,17].

Certain studies have indicated that the use of specific pharmacological interventions, such as BTA, venlafaxine, clomipramine, mianserin, and lidocaine, does not lead to a notable decrease in average pain intensity when compared to a placebo [9,11,14]. However, various studies have shown that certain medications can be beneficial in the treatment of AFP. According to Harrison (1997), subcutaneous administration of sumatriptan resulted in a significant decrease in pain scores after 120 min; however, this decrease was not large enough to be clinically beneficial [12]. Marbach et al. reported notable pain relief with high concentrations of intranasal cocaine [15]. Feinmann et al. (1983) showed that dothiepin notably decreases the severity of pain in individuals with psychogenic facial pain [17].

The effects of pharmacological therapies on depression differed among studies, with certain treatments demonstrating important advantages and others not. The reliable effectiveness of antidepressants, especially Tricyclic Antidepressants therapy (TCAs) and serotonin-norepinephrine reuptake inhibitor (SNRIs), in alleviating pain and enhancing quality of life aligns with their established mechanisms of action, which involve the modulation of pain pathways and the improvement of monoaminergic neurotransmission. Anticonvulsants, known for their ability to modulate neuronal excitability, also showed considerable pain relief, indicating a possible role in addressing neuropathic elements of AFP. The differences in results concerning depression and anxiety could indicate the intricate interaction between pain and psychological elements in AFP. Although certain patients might notice a lift in mood with successful pain management, others may need further psychological support to tackle underlying mental health issues. Future research should investigate the possible advantages of merging pharmacological treatment with psychotherapy or other non-drug interventions. Certain studies revealed no notable variances when comparing antidepressants such as venlafaxine and sumatriptan with alternative therapies such as placebos [11,12]. Additionally, Feinmann et al. demonstrated that dothiepin did not outperform placebo in alleviating depression, even though both therapies resulted in noteworthy enhancements in depression scores [17]. Loldrup et al. (1989) concluded that clomipramine was more effective than a placebo in reducing symptoms, demonstrating its ability to manage both pain and depression [14]. Moreover, Sharav et al. found that amitriptyline decreased depression scores in depressed individuals, but had no impact on non-depressed subjects, underscoring its particular efficacy for depressive symptoms [16]. This demonstrates the difficulty of addressing long-lasting depression, as medication may not always result in full recovery. These results highlight the importance of considering effectiveness and tolerability when selecting pharmacological therapies for depression. In general, pharmacological therapies for depression may work well for some individuals, but their effectiveness can differ greatly depending on the type of depression, particular drugs, and unique patient characteristics [3,6,7]. Jia et al. (2022) [21] performed an extensive review showing that duloxetine, a SNRIs, notably enhanced pain scores in individuals with Persistent Idiopathic Dentoalveolar Pain (PIDP) over a span of 12 weeks. Duloxetine was generally well-received, with few side effects noted. Similarly, Tu et al. (2019) [22] evaluated venlafaxine in their review and highlighted its promise in the management of PIDP. The study emphasized the drug’s effectiveness in patients who had failed traditional TCAs. Research from investigations like Jia et al. (2022) [21] and Tu et al. (2019) [22] emphasizes the possible advantages of SNRIs, anticonvulsants, and combination treatments. Understanding related orofacial pain conditions provides additional guidance for treatment approaches. A patient-centred and multidisciplinary strategy is crucial for enhancing results. Bendtsen et al. (2020) [23] examined gabapentinoids and tricyclic antidepressants as primary therapies for chronic facial pain. They pointed out that opioids ought to be avoided because of the potential for dependence and their limited effectiveness for chronic pain. Zakrzewska (2016) [24] emphasized the shortage of high-quality randomized controlled trials (RCTs) for AFP/PIFP, resulting in dependence on evidence from different neuropathic pain disorders. They proposed that drug treatments ought to be used alongside non-drug methods (e.g. cognitive-behavioral therapy) to achieve improved results. Xiao et al. (2024) [25] conducted a retrospective analysis of clinical data from patients diagnosed with PIDP to examine the safety and efficacy of venlafaxine in managing PIDP across various research centers. They stated that Venlafaxine could be effective and safe for treating PIDP, as 80.6% of patients found pain relief, and only 49.6% had side effects, primarily mild ones. They proposed that administering venlafaxine promptly after a PIDP diagnosis might increase the chances of achieving pain relief. Colloca (2019) [26] examined how the placebo effect significantly influences clinical outcomes. They demonstrated key aspects of placebo mechanisms that guide the methodology of clinical trials, the identification of new therapeutic targets, and the progress of personalized pain management. They typically noted that clinical trials involving pain groups frequently fail because interventions do not exceed placebo effects. This variation requires an individualized treatment approach, usually combining medication and therapy, to improve patient outcomes.

Harrison (1997) did not assess anxiety directly but observed decreases in anxiety through other outcomes [12]. This observation is consistent with the results of different studies on treating depression, which frequently indicate a decrease in anxiety as an additional advantage [12,14,15]. Furthermore, Marbach et al. (1988) suggested a decrease in anxiety through indirect observation, although they did not directly assess anxiety. This underscores the importance of considering secondary advantages when assessing the efficacy of pharmacological therapies for AFP. Improvements in depressive symptoms may help reduce anxiety, even if their impact on anxiety is not directly evaluated [15]. Similarly, Loldrup et al. (1989) discovered that mianserin was more effective in reducing anxiety symptoms than clomipramine and placebo [14]. A decrease in symptoms of depression is frequently linked to lower levels of anxiety, indicating that effective treatments for depression can indirectly improve anxiety levels. This shows that easily accepted treatments can help enhance overall mental well-being by decreasing anxiety.

The follow-up times varied among the studies owing to the different methodologies and durations of AFP treatment [9–11,14,16,17] Jamtoy et al. (2023) conducted a structured assessment 12 weeks after injection, followed by a washout phase of eight weeks, indicating that BTA might have extended benefits beyond the initial 12 weeks [9]. In contrast, Forssell et al. (2004) performed routine evaluations for a period of 10 weeks; however, they did not observe any notable variance in pain intensity decrease between venlafaxine and placebo [11]. In general, the different follow-up durations and results of these studies highlight the importance of personalized treatment strategies and further research to improve the management of AFP.

There was a range in the necessity for more painkillers in the different studies, indicating the efficacy of the main treatments. The study conducted by Jamtoy et al. (2023) found that ten participants requested extra pain medication on the day of their injections, suggesting that the initial use of BTA in the sphenopalatine ganglion may not have resulted in immediate pain relief [9]. In contrast, Forssell et al. (2004) found that analgesics were rarely used. This indicated that lidocaine, a local anaesthetic used in their study, effectively controls pain without the need for additional pain medication [11]. In general, these studies demonstrated differences in the necessity for extra pain medication, which was affected by how well the initial treatments worked and the specific types of pain being treated. Hence, the selection of the initial treatment and its efficacy are important factors in determining the need for additional painkillers.

Various side effects are associated with pharmacological treatments for AFP, which may affect patient adherence and treatment outcomes. To enhance the quality of life and treatment adherence, treatment decisions should be tailored to the specific needs of each patient, considering both efficacy and the possibility of side effects [9–11,13,27,28].

With comparable pain relief and better results in terms of medication use, healthcare utilization, and functional activity levels, comprehensive pain rehabilitation programs have become the better choice. Programmes for pain rehabilitation provide a thorough and efficient method of treating chronic pain. These programs are a potential option for people looking for long-term relief from chronic pain since they reduce pain to the same extent as standard therapies while producing better results in terms of medication use, healthcare consumption, and functional activities. To enhance patient outcomes and quality of life, multidisciplinary pain rehabilitation program expansion and support should be given top priority as healthcare systems continue to change.

## Conclusions

This systematic review showed that although pharmaceutical interventions are helpful, pain relief is not always achieved. Programs for pain rehabilitation, in particular, provide pain reduction that is equivalent to other modalities, but with superior results when it comes to the use of medications, healthcare utilization, and functional activities. This shows that AFP treatment may benefit from a multimodal strategy that combines pharmaceutical and nonpharmacological treatments.

## Supplementary Material

PRISMA_2020_checklist.docx

## Data Availability

The datasets used and analysed during the current study are available from the corresponding author upon reasonable request
